# Seasonal proteinuria changes in IgA nephropathy patients after proteinuria remission

**DOI:** 10.1371/journal.pone.0187607

**Published:** 2017-11-02

**Authors:** Koji Inagaki, Yoshinari Yasuda, Masahiko Ando, Ahmad Baseer Kaihan, Asaka Hachiya, Takaya Ozeki, Manabu Hishida, Takahiro Imaizumi, Takayuki Katsuno, Sawako Kato, Naotake Tsuboi, Shoichi Maruyama

**Affiliations:** 1 Department of Nephrology, Nagoya University Graduate School of Medicine, Nagoya, Japan; 2 Center for Advanced Medicine and Clinical Research, Nagoya University Hospital, Nagoya, Japan; Kawasaki Ika Daigaku, JAPAN

## Abstract

**Background:**

Proteinuria is a powerful prognostic factor for end-stage renal disease in IgA nephropathy (IgAN) patients. However, it is not known whether proteinuria exacerbations are related to seasonal changes.

**Methods:**

We retrospectively enrolled consecutive patients diagnosed with IgAN by kidney biopsy at our hospital between 2002 and 2014. Proteinuria remission was defined as urinary protein <0.3 g/gCr in two consecutive outpatient urinalyses and exacerbation as urinary protein ≥0.75 g/gCr. Four seasons were defined: spring (March–May), summer (June–August), autumn (September–November), and winter (December–February). We performed a multivariate analysis to identify factors associated with the second remission following a proteinuria exacerbation.

**Results:**

We analyzed 116 patients. Proteinuria remission and exacerbation occurred in 77, and 43 patients, respectively. The incidence of proteinuria exacerbation was significantly higher in autumn and winter than in spring and summer (p = 0.040). The cumulative second remission rate was significantly higher in patients with autumn and winter proteinuria exacerbation than in patients with spring and summer exacerbations (p = 0.0091). In multivariate analyses, exacerbation onset in autumn and winter (hazard ratio [HR], 3.51; 95% confidence interval [CI], 1.41–8.74) and intensive therapy (HR, 2.26; 95% CI, 1.05–4.88) were significantly associated with a second proteinuria remission.

**Conclusion:**

In IgAN patients in proteinuria remission, proteinuria exacerbation frequently occurred in autumn and winter. Exacerbations occurring in autumn and winter tended to remit early.

## Introduction

IgA nephropathy (IgAN) is the most common primary chronic glomerulonephritis worldwide [1]. In Japan, IgAN accounts for approximately 30% of all cases of primary glomerulonephritis [2]. However, IgAN has a poor renal prognosis with 40% of affected patients reaching end-stage renal disease (ESRD) within 20 years [3]. Proteinuria at the time of renal biopsy is a proven prognostic factor for adverse renal outcomes [4]. Immunosuppressive treatment and tonsillectomy could decrease proteinuria and improve renal prognosis [5–7].

Time-averaged proteinuria (TA-P) has been associated with ESRD [8]. Clinically significant proteinuria is usually defined as a baseline proteinuria and TA-P level ≥1.0 g/day [8]. Additionally, in a recent study, a TA-P between 0.5 and 1.0 g/day was a prognostic factor for kidney failure [9]. Although, many IgAN patients achieve complete remission with immunosuppressive agents, some experience proteinuria exacerbation after complete remission [10]. This finding suggests that proteinuria exacerbation is a cause of an unfavorable renal outcome as TA-P is elevated.

Proteinuria and albuminuria tend to occur more frequently in autumn and winter. Among early diabetic nephropathy patients, the urinary albumin/creatinine ratio was significantly higher in winter than in summer because systolic blood pressure (BP) was elevated in winter [11]. The incidence of childhood minimal change nephrotic syndrome (MCNS) was highest in autumn possibly due to an associated allergic predisposition [12]. However, it is not known whether IgAN-related proteinuria is affected by the seasons, and the management of proteinuria exacerbation occurring after remission in IgAN patients is controversial.

Therefore, we investigated the seasonal proteinuria changes in IgAN patients after proteinuria remission. Additionally, we investigated the factors associated with a second remission after proteinuria exacerbation.

## Materials and methods

### Study design and study subjects

This study was a retrospective cohort study in a single center. Study subjects were 145 consecutive IgAN patients aged 18 years and older, who were diagnosed by renal biopsy examination between 2002 and 2014 at Nagoya University Hospital because there were no detailed medical records before 2001. Twenty-seven patients with follow-up period less than 12 months were excluded from this study, and a total of 116 patients were analyzed. Institutional Review Board (IRB) committee was Nagoya university hospital and approval number was 1135/2015-0386. The committee approved this retrospective cohort study without written informed consent, but informed consent was obtained from almost all IgAN patients at the time of kidney biopsy.

### Definition of clinical parameters

Clinical parameters were collected from medical records at the time of renal biopsy. During follow-up, proteinuria was assessed by calculating the spot urine protein-creatinine ratio. Proteinuria remission was defined as urinary protein less than 0.3 g/gCr in two consecutive outpatient clinic urinalyses [13]. Proteinuria exacerbation was defined as urinary protein greater than 0.75 g/gCr [14]. The second remission was defined as urinary protein less than 0.3 g/gCr in two consecutive outpatient clinic urinalyses after proteinuria exacerbation. TA-P was calculated as the average urinary protein during each 6-month period from the time of kidney biopsy to proteinuria exacerbation [15]. The time- averaged mean arterial pressure (TA-MAP) was calculated as the average MAP measurements during the same 6-month period. Four seasons were defined as follows: spring (March–May), summer (June–August), autumn (September–November), and winter (December–February) [11]. Official record of mean outdoor temperature and mean humidity in Nagoya at the day of proteinuria exacerbation were obtained in the Japan Meteorological Agency website (http://www.data.jma.go.jp). The estimated glomerular filtration rate (eGFR) was calculated using the Japanese eGFR equation [eGFR = 194 x sCr^-1.094^ x age^-0.287^ x 0.739 (if female)] [16]. Hypertension was defined as blood pressure (BP) ≥140/90 mmHg or treatment with antihypertensive drugs. The observational period was at the end of August 2017.

### Histology

The renal biopsy specimens were stained with periodic acid-Schiff and Masson’s trichrome. Of116 patients, 111 had over eight glomeruli in their renal biopsy specimen. We evaluated pathological variables according to the Japanese histological classification (JHC) because in recent articles, proteinuria exacerbation and renal outcome were significant prognostic factors in Japanese IgAN patients according to the JHC, but not the Oxford classification [10, 17]. The JHC is a lumped system used to evaluate the severity of kidney biopsy samples. It includes four histological grades (HGs), HG 1, HG 2, HG 3, and HG 4 which correspond to <25, 25–49, 50–74, and ≥75% of the glomeruli, respectively. Histologically, lesions consist of cellular crescents, fi5% of the g crescents, fibrous crescents, segmental sclerosis, and global sclerosis [18].

### Treatment

Methylprednisolone (mPSL) pulse therapy, according to the protocol reported by Pozzi, et al, consisted of three consecutive days of mPSL pulse therapy three times at 2-month intervals, combined with oral prednisolone (PSL), 0.5 mg/kg every other day for six months [5, 6]. Intensive therapy after proteinuria exacerbation was defined as the addition or an increased dose of immunosuppressive drugs, including PSL, or tonsillectomy. Supportive therapy after proteinuria exacerbation was defined as observation or addition or increased doses of renin-angiotensin system blockers (RASBs).

### Statistical analysis

Normally distributed variables are expressed as means ± standard deviation (SD) and were compared using Student’s t-test. Nonparametric variables are expressed as medians and interquartile ranges (IQRs) and were compared using the Mann–Whitney *U* test. Categorical variables are expressed as numbers and proportions and were compared using Fisher’s exact test. The rate of survival without a second remission was analyzed by the Kaplan-Meier method and the log-rank test. The starting point of follow-up in the survival analysis was defined as proteinuria exacerbation. Univariate Cox regression was used to determine factors predicting the second remission. Intensive therapy was tested as a time-dependent variable. Significant variables shown in univariate analysis were further examined in multivariate analysis with well-known renal prognostic factors of TA-MAP, eGFR, TA-P, U-RBC and JHC. [6, 7, 8, 9, 18] These results are expressed as hazard ratios (HRs) and 95% confidence intervals (CIs). P values <0.05 were considered statistically significant. All statistical analyses were performed using EZR (Saitama Medical Center, Jichi Medical University, Saitama, Japan), which is a graphical user interface for R (The R Foundation for Statistical Computing, Vienna, Austria) [19].

## Results

### Clinical and histological characteristics at the time of kidney biopsy and treatment

The mean patient age was 37.9 ± 14.3 years, and 65 (56.0%) were female. The median proteinuria was 0.96 g/day (IQR, 0.59–1.67 g/day), and the mean eGFR was 74.9 ± 24.9 ml/min/1.73 m^2^. HGs 1, 2, 3, and 4 were observed in 58 (52.3%), 33 (29.7%), 18 (16.2%), and 2 (1.8%) patients, respectively.

RASBs were prescribed to 90 of 116 IgAN patients (77.6%). Oral PSL was prescribed to 85 (73.3%) patients. The median period between kidney biopsy and the initiation of oral PSL was 31 days (IQR, 8–88 days). Eighty- two (96.5%) patients who received oral PSL were treated with mPSL pulse therapy. Twenty-seven (23.3%) patients underwent tonsillectomy. The median period between kidney biopsy and tonsillectomy was 219 days (IQR, 69–595 days).

During a median follow-up of 74.4 months (IQR: 45.5–114.8 months), 89 (76.7%) patients achieved proteinuria remission. After a median remission period of 26.0 months (IQR, 8.0–40.7), proteinuria exacerbation occurred in 50 (56.2%) patients. Median urine measurement interval was 1.64 months (IQR, 1.39–2.07 months) between proteinuria remission and exacerbation.

The baseline characteristics at the time of kidney biopsy and the initial proteinuria remission between the exacerbation and no exacerbation groups are shown in Table 1. At the time of kidney biopsy, the eGFR was significantly lower and proteinuria was significantly higher in the exacerbation group than in the no exacerbation group. However, JHC and hypertension did not differ significantly between the two groups. At the first therapy and the initial proteinuria remission, there were no differences between the two groups.

**Table 1:** Characteristics of patients who did and did not experience proteinuria exacerbation after proteinuria remission at the time of kidney biopsy and initial proteinuria remission.

	All patients (n = 89)	Exacerbation group (n = 50)	No exacerbation group (n = 39)	P value
**Characteristics of the time of kidney biopsy**				
Age, years	36.1 ± 13.3	37.2 ± 13.8	34.6 ± 12.7	0.36
Male/female	36 (40.5) / 53 (59.5)	16 (32.0) / 34 (68.0)	20 (51.3) /19 (48.7)	0.083
BMI, kg/m^2^	21.6 ± 3.17	21.6 ± 3.21	21.7 ± 3.16	0.91
Diabetes mellitus	3 (3.4)	2 (4.0)	1 (2.6)	1
Hypertension	29 (32.6)	19 (38.0)	10 (25.6)	0.26
SBP, mmHg	120.5 ± 16.5	120.3 ± 16.8	120.8 ± 16.3	0.89
DBP, mmHg	71.6 ± 11.7	72.4 ± 12.3	70.6 ± 11.0	0.49
eGFR, ml/min /1.73 m^2^	79.2 ± 24.2	74.5 ± 23.1	85.4 ± 24.4	0.034
Serum IgA, mg/dL	300 [239–358]	313 [246–387]	271 [238–331]	0.10
IgA/C3 ratio	3.06 [2.48–3.90]	3.24 [2.53–3.99]	2.88 [2.30–3.46]	0.11
UPE, g/day	0.92 [0.49–1.66]	1.03 [0.62–1.70]	0.81 [0.42–1.36]	0.12
UPE, g/gCr	1.12 [0.47–1.89]	1.39 [0.75–2.17]	0.73 [0.39–1.64]	0.033
U-RBC ≥30/HPF	41 (46.1)	23 (46.0)	18 (46.2)	1
JHC Ⅰ /Ⅱ/Ⅲ/Ⅳ	49(57.0)/23(26.7)/12(14.0)/2(2.3)	25(52.1)/15(31.2)/7(14.6)/1(2.1)	24(63.2)/8(21.1)/5(13.2)/1(2.6)	0.71
**Characteristics of the first therapy before proteinuria remission**				
RASBs	64 (71.9)	39 (78.0)	25 (64.1)	0.16
Oral PSL	70 (78.7)	41 (82.0)	29 (74.4)	0.44
The period between kidney biopsy and the initiation of PSL, days	32 [8–80]	30 [8–72]	38 [9–128]	0.28
mPSL therapy	68 (76.4)	40 (80.0)	28 (71.8)	0.45
Tonsillectomy	20 (22.5)	9 (18.0)	11 (28.2)	0.31
The period between kidney biopsy and tonsillectomy, days	232 [58–592]	244 [49–494]	219 [69–734]	0.68
**characteristics of the initial proteinuria remission**				
SBP, mmHg	116.4 ± 14.8	115.3 ± 15.5	117.8 ± 14.0	0.43
DBP, mmHg	69.6 ± 10.7	68.9 ± 11.6	70.5 ± 9.73	0.50
UPE, g/gCr	0.2 [0.15–0.25]	0.2 [0.16–0.25]	0.2 [0.15–0.25]	0.99
U-RBC≥ 5/HPF	46 (51.7)	22 (44.0)	24 (61.5)	0.14
The period between initial therapy and proteinuria remission, days	177 [58–440]	211 [86–479]	165 [41–395]	0.29

Values are presented as mean (± SD), median [IQR], and Numbers (%). Abbreviations: BMI, body mass index; SBP, systolic blood pressure; DBP, diastolic blood pressure; eGFR, estimated glomerular filtration rate; UPE, urinary protein excretion; U-RBC, urinary red blood cell sediments; HPF, high power field; JHC, Japanese histological classification; RASBs, renin-angiotensin system blockers; PSL, prednisolone; mPSL, methylprednisolone

### The incidence of proteinuria exacerbation

Proteinuria exacerbation occurred in nine patients (18.0%) in spring, five (10.0%) in summer, 13 (26.0%) in autumn, and 23 (46.0%) in winter, respectively. The incidence of proteinuria exacerbation was significantly higher in autumn and winter than in spring and summer (p = 0.040).

### The characteristics of the proteinuria exacerbation in two seasonal periods

Table 2 shows the characteristics of patients who experienced proteinuria exacerbation in two seasonal periods (spring / summer and autumn / winter). Mean outdoor temperature at the day of proteinuria exacerbation was significantly lower in the autumn and winter proteinuria exacerbation group than in the spring and summer exacerbation groups. The rates of RASB use and the previous history of hypertension were higher in the winter and autumn proteinuria exacerbation group than in the spring and summer exacerbation groups, but this difference was not statistically significant. Age, antecedent infection, eGFR, TA-P, TA- MAP, urinary red blood cell sediment (U-RBC), mean humidity and JHC were not significantly different between the two groups.

**Table 2:** Characteristics of patients who experienced proteinuria exacerbation in two combined seasonal periods.

	Spring and summer (n = 14)	Autumn and winter (n = 36)	P value
**Characteristic at kidney biopsy**			
Hypertension	3 (21.4)	16 (44.4)	0.20
BMI	21.5 ± 2.80	21.6 ± 3.39	0.92
Serum IgA, mg/dL	324.5 [241.8–486.8]	313.0 [261.5–364.0]	0.67
IgA/C3 ratio	3.53 [2.58–4.78]	3.13 [2.56–3.90]	0.49
JHC Ⅰ / Ⅱ+Ⅲ+Ⅳ	9(64.3)/5(35.7)	16(47.1)/18(52.9)	0.35
**Characteristic at proteinuria exacerbation**			
Age, years	40.9 ± 15.2	40.8 ± 13.3	0.99
Male/Female	3/11 (21.4/78.6)	13/23 (36.1/63.9)	0.50
Antecedent infection	4 (28.6)	6 (16.7)	0.44
Usage of RASB	6 (42.9)	24 (66.7)	0.20
Usage of oral PSL	2 (14.3)	7 (19.4)	1
SBP, mmHg	116.6 ± 19.9	118.8 ± 14.9	0.68
DBP, mmHg	74.6 ± 10.6	73.5 ± 14.1	0.79
MAP, mmHg	88.6 ± 12.7	88.6 ± 13.4	0.99
TA-MAP, mmHg	84.7 ± 9.40	85.2 ± 9.53	0.88
eGFR, ml/min/1.73m^2^	70.7 ± 20.3	70.0 ± 21.8	0.92
UPE, g/gCr	0.93 [0.86–1.12]	1.03 [0.84–1.22]	0.75
TA-P, g/gCr	0.52 [0.37–0.70]	0.49 [0.38–0.75]	0.90
Remission duration, month	22.1 [9.84–48.9]	26.1 [7.59–34.3]	0.66
U-RBC≥ 5/HPF	8 [57.1]	16 [44.4]	0.53
Mean outdoor temperature, °C	16.0 ± 8.44	9.31 ± 6.64	0.005*
Mean humidity, %	67.9 ± 12.9	63.3 ± 12.2	0.24
Intensive therapy	7 (50.0)	14 (38.9)	0.53
Second remission	6 (42.9)	28 (77.8)	0.040*

Values are presented as mean (± SD), median [IQR], and Numbers (%). Abbreviations: BMI, body mass index; JHC, Japanese histological classification; RASBs, renin-angiotensin system blockers; PSL, prednisolone; SBP, systolic blood pressure; DBP, diastolic blood pressure; MAP, mean arterial pressure; TA-MAP, time-averaged arterial pressure; eGFR, estimated glomerular filtration rate; UPE, urinary protein excretion; TA-P, time-averaged proteinuria; U-RBC, urinary red blood cell sediments; HPF, high power field.

* p < 0.05

### Predictive factors for the second remission

Of the 50 patients with proteinuria exacerbation, 34 (68.0%) patients achieved a second remission. Twenty-one (42.0%) patients were treated with intensive therapy at median 106 days (IQR, 63–449 days) after proteinuria exacerbation. Of the 21 patients who received intensive therapy, 17 patients (81.0%) had immunosuppressive drugs including PSL, cyclosporine, and/or mizoribine added or increased; 2 patients (9.5%) underwent tonsillectomy, and 2 patients (9.5%) underwent steroid in combination with tonsillectomy. The rate of intensive therapy use was not significantly different between spring/summer and autumn/winter groups (Table 2).

**Fig 1:**
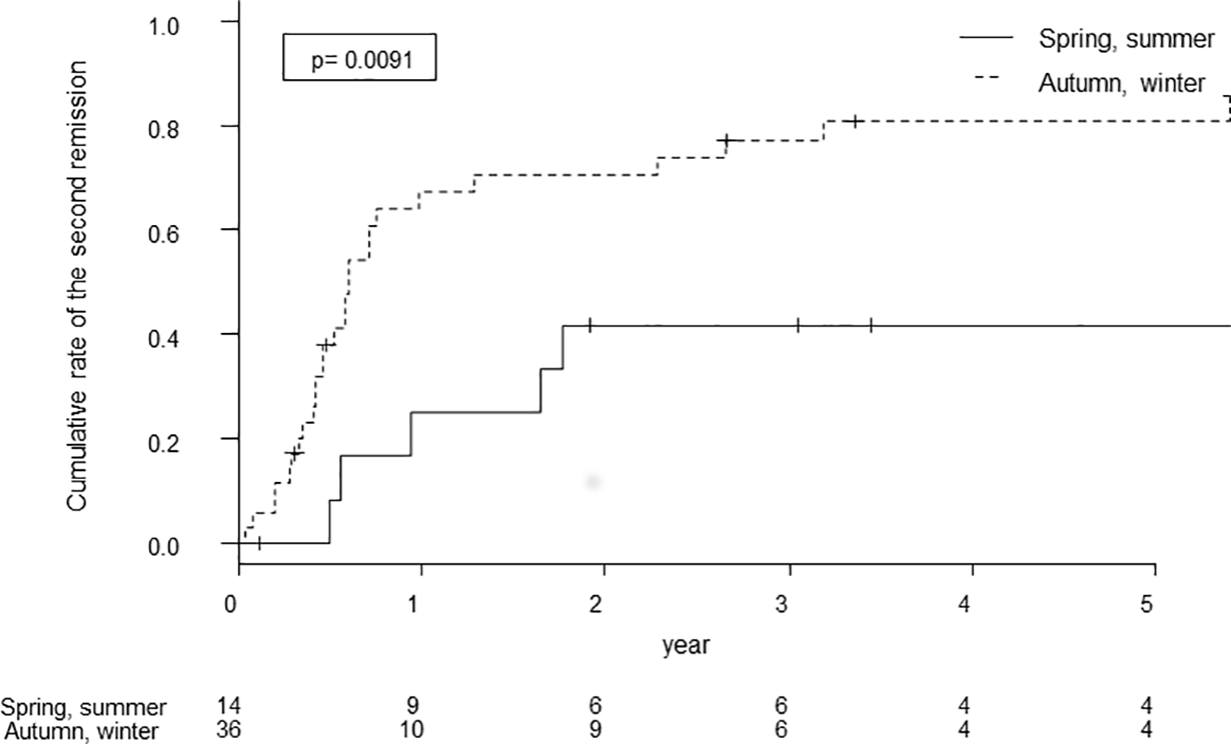
Kaplan–Meier analyses of the second remission after proteinuria exacerbation in two combined seasonal periods.

The cumulative second remission rate was significantly higher in patients with proteinuria exacerbation in autumn and winter than in patients with spring and summer exacerbations (log-rank test, p = 0.0091; Fig 1). Univariate Cox proportional hazards regression analysis showed proteinuria exacerbation in autumn and winter (HR, 3.08; 95% CI, 1.26–7.52) and intensive therapy (HR, 2.10; 95% CI, 1.03–4.27) were predictors for a second remission (Table 3). However, mean outdoor temperature at the day of proteinuria exacerbation was not a predictor for the second remission. Multivariate analysis was carried out using TA-MAP, eGFR, TA-P, U-RBC, intensive therapy, season, and JHC. Proteinuria exacerbation in autumn and winter (HR, 3.51; 95% CI, 1.41–8.74) and intensive therapy (HR, 2.26; 95% CI, 1.05–4.88) were significant predictors for a second remission (Table 3).

**Table 3:** Univariate and multivariate Cox regression analyses of factors associated with a second remission (Intensive therapy was tested as a time-dependent variable).

Variables	Univariate Model		Multivariate Model	
	HR [95% CI]	P value	HR [95% CI]	P value
Hypertension (kidney biopsy)	0.99 [0.49–2.04]	0.99		
IgA/C3 ratio≥ 3 (kidney biopsy)	0.99 [0.49–2.00]	0.98		
Age (per 10years.)	0.97 [0.76–1.24]	0.83		
Sex (male)	1.04 [0.49–2.20]	0.92		
Antecedent infection	1.45 [0.65–3.24]	0.36		
Usage of RASBs	0.64 [0.32–1.27]	0.20		
Usage of PSL	1.04 [0.42–2.53]	0.94		
MAP (per 10mmHg)	1.17 [0.90–1.51]	0.24		
TA-MAP (per 10mmHg)	1.23 [0.84–1.82]	0.29	1.29 [0.83–2.01]	0.26
eGFR≤ 60 ml/min/1.73m^2^	1.20 [0.57–2.54]	0.63	1.08 [0.42–2.75]	0.87
UPE≥ 1g/gCr	0.83 [0.41–1.67]	0.60		
TA-P≥ 0.5g/gCr	0.84 [0.42–1.66]	0.62	0.81 [0.39–1.67]	0.57
U-RBC≥ 5/HPF	1.07 [0.54–2.10]	0.85	0.80 [0.37–1.74]	0.57
Remission duration (per 1 month)	0.99 [0.98–1.01]	0.54		
JHC Ⅱ+Ⅲ+Ⅳ	0.99 [0.50–1.98]	0.98	0.69 [0.33–1.45]	0.33
Season (autumn, winter)	3.08 [1.26–7.52]	0.013*	3.51 [1.41–8.74]	0.0069*
Mean outdoor temperature	0.99 [0.95–1.04]	0.75		
mean humidity (per 10%)	1.03 [0.79–1.33]	0.83		
Intensive therapy	2.10 [1.03–4.27]	0.042*	2.26 [1.05–4.88]	0.038*

Abbreviations: HR, hazard ratio; CI, confidence interval; RASBs, renin-angiotensin system blockers; PSL, prednisolone; MAP, mean arterial pressure; TA-MAP, time-averaged mean arterial pressure; eGFR, estimated glomerular filtration rate; UPE, urinary protein excretion; TA-P, time-averaged proteinuria; U-RBC, urinary red blood cell sediments; HPF, high power field; HG, histological grade; JHC, Japanese histological classification

* p < 0.05

### Supportive therapy after proteinuria exacerbation

Of 43 patients who experienced proteinuria exacerbation and were followed up for more than one year, 30 (69.8%) had received supportive therapy for one year. Among seven patients with spring and summer proteinuria exacerbations who received supportive therapy, none had achieved a second remission within one year. On the other hand, of 23 patients who received supportive therapy for autumn and winter proteinuria exacerbations, 15 (65.2%) had achieved a second remission within one year. These differences in response to supportive therapy between the two combined seasonal groups was statistically significant (p = 0.0063).

## Discussion

We retrospectively studied IgAN patients and found that proteinuria remission frequently presented with proteinuria exacerbation in autumn and winter. Reports indicate that patients with diabetic nephropathy and childhood MCNS tend to experience proteinuria and albuminuria exacerbations in autumn and winter [11, 12]. However, no investigator has reported an association between IgAN and seasonal proteinuria exacerbation. To our knowledge, this is the first report demonstrating a relationship between season and IgAN.

Although the mechanisms underlying the increased incidence of proteinuria exacerbation in autumn and winter are poorly understood, two explanations may account for the relationship between IgAN and season. First, we hypothesize hypertension might result in proteinuria exacerbation because the presence of microscopic hematuria was not significantly different between the two- combined seasonal periods. In general, BP rises in winter due to vasoconstriction and falls in summer due to vasodilation because of the change in ambient temperature [20]. Additionally, Berthoux et al. reported that control of hypertension reduced the risk of death or dialysis in IgAN patients [21]. Second, we thought that antecedent infection might result in proteinuria exacerbation. IgAN was sometimes preceded by upper respiratory infection, and these tended to occur more frequently in winter [22]. However, we could not identify a relationship between seasonal proteinuria change and BP or infection. Two aspects of our study may explain this fact. First, BPs were measured in the clinic only. Anderson reported that approximately 26% of chronic kidney disease patients with normal BP in the clinic have high BP at home [23]. Lin et al. reported that the urine protein/creatinine ratio is directly associated with nocturnal hypertension among IgAN patients [24]. Second, our study was a retrospective study and, thus, dependent on medical records that may not have fully recorded the incidence of infection in our patients. Thus, prospective and large population studies, including home BP measurement and precise documentation of infection is needed.

Our study also demonstrated that patients with proteinuria exacerbation in autumn and winter tended to achieve a second remission. In this study, mean outdoor temperature and mean humidity were analyzed as alternative meteorology information. Although outdoor temperature in Japan was lower in autumn and winter than in spring and summer, mean outdoor temperature at the day of proteinuria exacerbation was not significantly associated with a second remission. Moreover, mean humidity at the day of proteinuria exacerbation was not different in autumn and winter than in spring and summer. In hypertensive patients, the association of indoor temperature was stronger than that of outdoor temperature relative with regard to sBP [25]. Additionally, the correlation between indoor and outdoor temperature decreased on cold days [25]. Therefore, home temperature would be candidate for further study why the second remission was frequently occurred in autumn and winter among IgAN patients with proteinuria exacerbation.

Interestingly, among patients who experienced proteinuria exacerbation in autumn and winter and were given supportive therapy alone, approximately 65% achieved a second remission. However, in patients who experienced proteinuria exacerbation in spring and summer and received supportive therapy alone, none achieved the second remission. No patient who experienced proteinuria exacerbation developed acute kidney injury (AKI) or nephrotic syndrome in this study. Patients with IgAN and AKI or nephrotic syndrome have a poor renal outcome and will need immunosuppressive therapy. [26, 27]. In patients who develop proteinuria exacerbation in autumn or winter, intensive therapy including immunosuppression agents and tonsillectomy might not be needed in the absence of AKI or nephrotic syndrome.

This study has three limitations. First, this was a retrospective study, and the medical records might not have been complete. Second, the study population was small. Third, we could not identify the mechanism underlying seasonally-related proteinuria changes with certainty. Nonetheless, we successfully demonstrated the relationship between seasonal proteinuria change and IgAN. Additionally, we demonstrated that proteinuria exacerbation in autumn and winter tended to remit early.

## Conclusions

In conclusion, in IgAN patients in proteinuria remission, the incidence of proteinuria exacerbation is higher in autumn and winter than in spring and summer. When proteinuria exacerbation occurs in autumn and winter, it is better to follow the patient without intensive therapy.

## Supporting information

S1 Fig Proteinuria trajectory of 50 patients who experienced proteinuria exacerbation from the initial remission to end of follow-up. (TIF)

S2 Fig Seasonal time-averaged proteinuria (seasonal TA-P) of 50 patients who experienced proteinuria exacerbation. (TIF)

S1 Dataset The anonymous dataset of 116 IgAN patients at the time of kidney biopsy. (XLSX)

S2 Dataset The anonymous dataset of 50 patients with proteinuria exacerbation. (XLSX)
