# Supplementary figures and images for: Seasonal proteinuria changes in IgA nephropathy patients after proteinuria remission
Source: PLoS One. 2017 Nov 2;12(11):e0187607. doi: 10.1371/journal.pone.0187607 (PMC5667876; doi:10.1371/journal.pone.0187607)

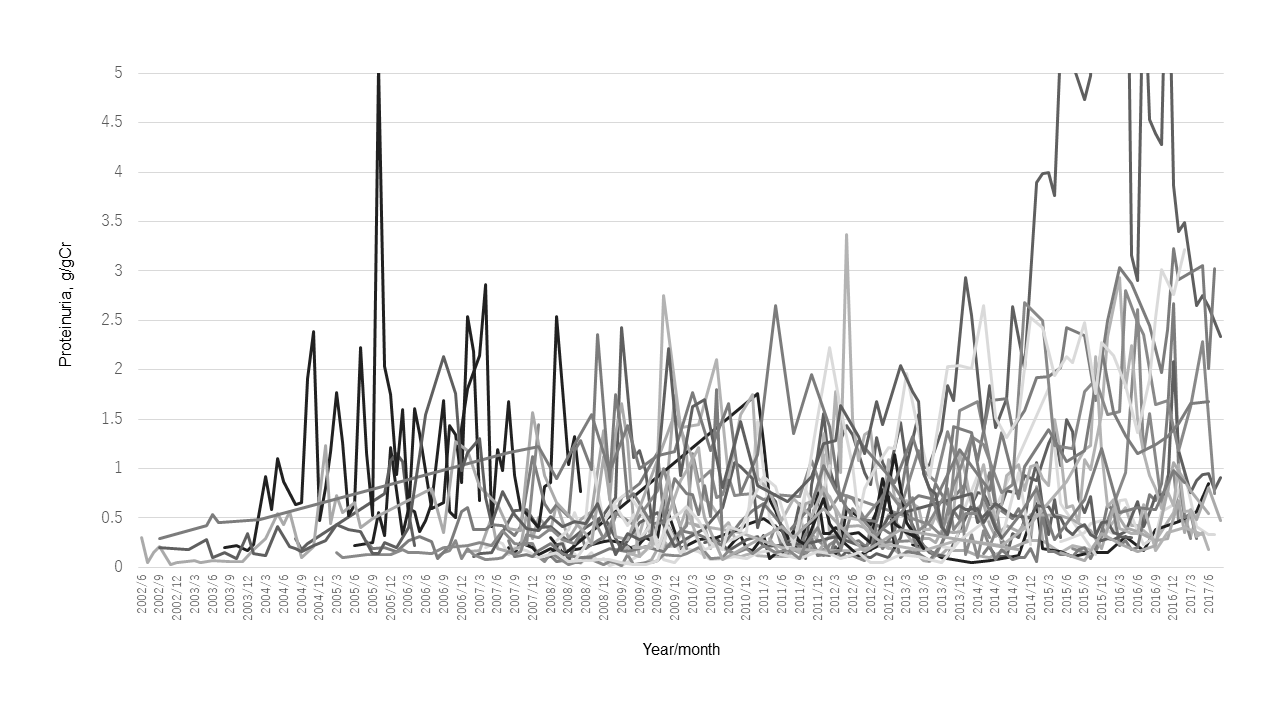

Supplement: S1 Fig — (TIF) [file pone.0187607.s001.tif]

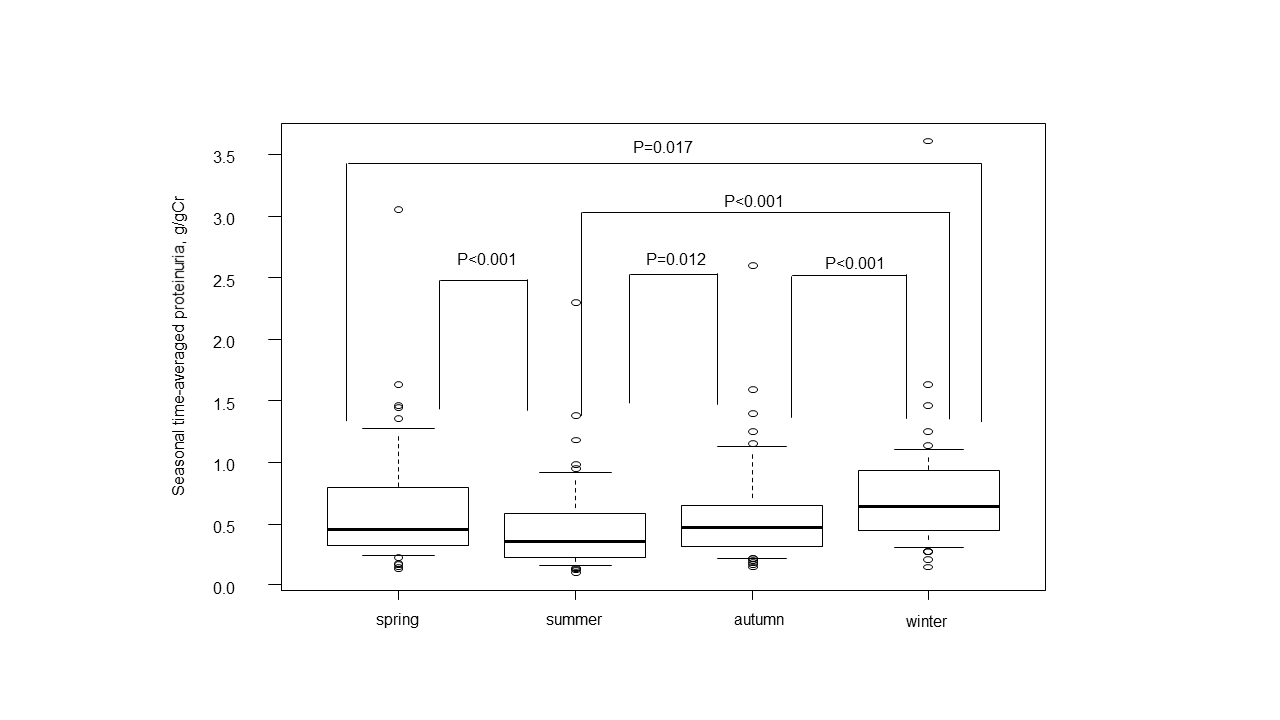

Supplement: S2 Fig — (TIF) [file pone.0187607.s002.tif]
